# A history of asthma is associated with susceptibility to hidradenitis suppurativa: a population-based longitudinal study

**DOI:** 10.1007/s00403-023-02693-4

**Published:** 2023-08-29

**Authors:** Khalaf Kridin, Wesal Shihade, Orly Weinstein, Lilach Zoller, Erez Onn, Arnon Cohen, Efrat Solomon-Cohen

**Affiliations:** 1 Unit of Dermatology and Skin Research Laboratory, Galilee Medical Center, Nahariya, Israel; 2 Azrieli Faculty of Medicine, Bar-Ilan University, Safed, Israel; 3 Lübeck Institute of Experimental Dermatology, University of Lübeck, University of Lübeck, Ratzeburger Allee 160, 23562 LübeckLübeck, Germany; 4 School of Public Health, University of Haifa, Haifa, Israel; 5 Clalit Health Services, Tel-Aviv, Israel; 6 Siaal Research Center for Family Medicine and Primary Care, Faculty of Health Sciences, Ben-Gurion University of the Negev, Beer Sheva, Israel; 7 Baruch Padeh Medical Center, Poriya, Israel; 8 Division of Dermatology, Rabin Medical Center, Petah-Tiqva, Israel; 9 Sackler School of Medicine, Tel Aviv University, Tel Aviv, Israel

**Keywords:** Hidradenitis suppurativa, Asthma, Risk, Association

## Abstract

The association of hidradenitis suppurativa (HS) and asthma remains to be investigated. To assess the bidirectional association between HS and asthma. A population-based study was conducted to compare HS patients (*n* = 6779) with age-, sex-, and ethnicity-matched control subjects (*n* = 33,259) with regard to the incidence of new onset and the prevalence of preexisting asthma. Adjusted hazard ratios (HRs) and adjusted odds ratios (ORs) were calculated. The prevalence of preexisting asthma was higher in patients with HS relative to controls (9.6% vs. 6.9%, respectively; *P* < 0.001). The odds of HS were 1.4-fold greater in patients with a history of asthma (fully adjusted OR 1.41; 95% CI 1.27–1.55; *P* < 0.001). The incidence rate of new-onset asthma was estimated at 9.0 (95% CI 6.3–12.7) and 6.2 (95% CI 5.1–7.5) cases per 10,000 person-years among patients with HS and controls, respectively. The risk of asthma was not statistically different in patients with HS and controls (fully adjusted HR 1.53; 95% CI 0.98–2.38; *P *= 0.062). Relative to other patients with HS, those with HS and comorbid asthma were younger at the onset of HS (30.7 [14.7] vs. 33.3 [15.1], respectively; *P* < 0.001) and had a comparable risk of all-cause mortality (adjusted HR 0.86; 95% CI 0.44–1.68; *P* = 0.660). A history of asthma confers susceptibility to subsequent development of HS. This observation is of importance for clinicians managing both patients with HS and asthma. Further research is warranted to elucidate the pathomechanism underlying this finding.

**Supplementary Information:**

The online version contains supplementary material available at 10.1007/s00403-023-02693-4.

## Introduction

Hidradenitis suppurativa (HS) is a chronic inflammatory follicular disorder manifesting with recurrent and frequent painful deep-seated nodules, mainly of the skin folds. In advanced stages, draining sinus tract formation is accompanied by abscesses, fistulae, and scarring [1]. Since HS is a chronic disease and frequent relapses are common, it confers a longstanding impact on the patient’s quality of life and impairs social, occupational, and psychological domains [2, 3]. In the general population, HS affects at least three times more women than men, and it generally occurs after puberty with a prototypical onset during the second or third decades of life [1]. The exact prevalence of HS is uncertain, with ranges from 0.1% to 2% of the general population based on previous reports, and it is probably growing and higher than this [1–3].

The etiology and pathogenesis of HS are yet to be fully understood, with most recent theories suggesting a multifactorial origin consisting of genetic and environmental factors, immune system interactions, lifestyle habits, hormonal status, and microbiota [4]. HS was previously described as an inflammatory disease of the apocrine sweat glands. However, recent theories for its pathogenesis suggest hyperkeratosis of the follicular epithelium as the initial feature of the process, causing occlusion of the apocrine glands with subsequent follicular rupture (Nguyen TV)^,^ [6]. [5, 6]. This, in turn, leads to an increased release of proinflammatory cytokines, such as interleukin (IL)-1 beta, IL-12, IL-23, and tumor necrosis factor-alpha (TNF-a), both in perilesional skin biopsies and in the blood [7].

Recently reported a statistically significant association between HS and atopic dermatitis, a widespread inflammatory dermatosis with an allergic pathogenic component [9]. Considerable overlap exists between the immune cells and cytokine profile present in HS lesions and those implicated in the pathogenesis of allergic diseases [10, 11]. Some HS patients even manifest with high circulating immunoglobulin E levels [12] and itch [13]. Thus, it is highly intriguing to assess the epidemiological relationship between HS and asthma, one of the most frequently encountered allergic diseases worldwide [14]. This association was sparsely investigated in the past, and the current literature is relatively inconsistent about it [8, 15, 16]. Aiming at closing this gap in our knowledge, the current study investigates the bidirectional relationship between HS and asthma using a large-scale population-based study population.

## Methods

### Study design and database

Utilizing a large population of patients with HS, the current population-based study aimed to evaluate the bidirectional association between HS and asthma, that is, to estimate (i) the odds of HS after asthma and (ii) the risk of asthma after HS. To fulfill the first endpoint, a case–control study design assessing the prevalence of preexisting asthma in patients with HS and controls was followed. The second endpoint was answered by a retrospective cohort study design which followed patients with HS and controls longitudinally to estimate the incidence of new-onset asthma. The study was approved by the institutional review board (IRB) of Ben-Gurion University in accordance with the Declaration of Helsinki (approval code: 0212-17-COM).

The computerized database of Clalit Health Services (CHS) was the source of the current study findings. CHS is the largest healthcare maintenance organization (HMO) in Israel, providing a broad array of private and public healthcare services for 4,540,768 enrollees as of October 2018. CHS enrollees represent 52% of the general Israeli population and mirror the composition of the general Israeli society in light of the National Health Insurance law mandating all residents in the country to join one of the four official HMOs. This database continuously extracts data from several sources covering both ambulatory and hospitalized care settings, which is eventually documented in the enrollee’s medical file. Given that CHS is characterized by free access to healthcare service, inclusive certification, and negligible loss to follow-up, it is highly compatible with providing valid and reliable epidemiological data [17].

### Study population and definition of main variables and covariates

To recruit the current study population, the computerized database of CHS was systematically screened for enrollees with a diagnosis of HS between January 2000 and December 2018. Patients were subject to inclusion if at least one of the eligibility criteria was met: (i) registration of HS-specific diagnostic code by a CHS board-certified dermatologist, or (ii) documentation of a diagnosis of HS in discharge letters from inpatient dermatological wards.

A control group comprising up to 5 individuals per case of HS was additionally enlisted. Controls were matched to cases upon sex, age, and ethnicity. A date of ‘pseudodiagnosis’ was assigned to each one of the control subjects. The latter corresponds to the date on which the diagnosis of HS was documented in the respective case. The diagnosis of asthma was based on its documentation in the chronic disease registry of CHS. Thereby, it was delivered by pulmonologists, pediatricians, or internal medicine specialists and is subsequently cross-checked manually by the managing general practitioner.

Study outcomes were controlled for underlying comorbidities as assessed by the Charlson comorbidity index (CCI), an epidemiological scale evaluating the severity of comorbidities among study participants. The latter was proved reliable in predicting mortality [18 new] and is widely implemented in epidemiological studies. To avoid over-adjustment bias, a modified version of CCI excluding respiratory diseases was adopted in the current analysis. Outcome measures were additionally adjusted for body mass index (BMI) and demographic variables. The death date of study participants was ascertained by crosslinking the study cohort with the National Registry of Deaths Database. In the mortality analysis, all eligible patients were followed up from the onset of HS until December 31, 2018, or death, whichever had occurred earlier.

### Statistical analysis

Baseline characteristics were described by means and standard deviations (SD)s for continuous variables and percentages for categorical variables. The comparison between subgroups was performed using the chi-square test and t-test for categorical and continuous variables, respectively.

In the case–control study design, logistic regression was utilized to calculate odds ratios (ORs) and 95% confidence intervals (CIs). Individuals who developed HS before the time of their asthma diagnosis were excluded from this particular In the retrospective cohort study design, incidence rates of asthma were calculated for both HS patients and controls and expressed as the number of events per 10,000 person-years. The incidence of these outcomes was calculated merely for individuals without a history of asthma prior to the study initiation (diagnosis of HS or ‘pseudodiagnosis’ of controls). Hazard ratios (HRs) and 95% CIs for the risk of new-onset asthma were acquired by the use of Cox regression models. The cumulative survival of HS patients with and without asthma was calculated using Kaplan–Meier method and compared between the subgroups by stratified log-rank test. Two-tailed P-values less than 0.05 were considered statistically significant. All statistical analyses were performed using SPSS software, version 25 (SPSS, Armonk, NY: IBM Corp).

## Results

### Characteristics of the study population

The current study population consisted of 6779 patients with HS and 33,259 age-, sex-, and ethnicity-matched control individuals. The mean (SD) age at the diagnosis of HS was 33.1 (15.1) years, and 4071 (60.1%) were females. The mean BMI and the lifetime prevalence of smoking, diabetes mellitus, hyperlipidemia, and hypertension were significantly higher in cases than in controls. The demographic and clinical features of the study participants are delineated in Table 1**.**

**Table 1:** Descriptive characteristics of the study population

Characteristic	Patients with HS (*N* = 6779)	Controls (*N* = 33,259)	*P* value
Age, years			
Mean (SD)	33.1 (15.1)	33.1 (15.1)	0.817
Median (range)	30.0 (0.6–88.7)	30.0 (0.6–88.8)	
Sex, *N* (%)			
Male	2708 (39.9%)	13,347 (40.1%)	0.780
Female	4071 (60.1%)	19,913 (59.9%)	
Ethnicity, *N* (%)			
Jews	5607 (82.7%)	27,413 (82.4%)	0.768
Arabs	1172 (17.3%)	5846 (17.6%)	
BMI, mg/kg^2^			
Mean (SD)	27.2 (6.5)	24.7 (5.6)	** < 0.001**
Smoking, *N* (%)	3591 (53.0%)	11,469 (34.5%)	** < 0.001**
Diabetes Mellitus, *N* (%)	698 (10.3%)	2236 (6.7%)	** < 0.001**
Hyperlipidemia, *N* (%)	1972 (29.1%)	7690 (23.1%)	** < 0.001**
Hypertension, *N* (%)	853 (12.6%)	3220 (9.7%)	** < 0.001**
Charlson comorbidity score			
Mean score (SD)	0.5 (1.2)	0.4 (1.0)	** < 0.001**
None (0)	4891 (72.1%)	26,375 (79.3%)	** < 0.001**
Moderate (1–2)	1469 (21.7%)	5531 (16.6%)	** < 0.001**
Severe (≥ 3)	419 (6.2%)	1355 (4.1%)	** < 0.001**

*HS* hidradenitis suppurativa, *N* number, *SD* standard deviation, *BMI* body mass index

### The odds of hidradenitis suppurativa after asthma (case–control design)

The prevalence of preexisting asthma was significantly higher among patients with HS relative to controls (9.6% vs. 6.9%, respectively; *P* < 0.001). Therefore, the odds of HS were 1.4-fold higher in individuals with a history of asthma (OR 1.43; 95% CI 1.30–1.57). In an age-, sex-, and, ethnicity-stratified analysis, the association of asthma with subsequent HS was greater among individuals older than 30 years (OR 1.48; 95% CI 1.28–1.71; *P* < 0.001), females (OR 1.50; 95% CI 1.34–1.69; *P* < 0.001), and Arabs (OR 1.52; 95% CI 1.17–1.98; *P* = 0.002; Table 2). When patients were stratified by the latency between asthma and HS, the strongest association was found in subjects in whom the diagnosis of asthma preceded that of HS by more than 10 years (OR 1.44; 95% CI 1.28–1.62; *P* < 0.001; Table 2).

**Table 2:** The odds of hidradenitis suppurativa in patients with a preexisting diagnosis of asthma stratified by age, gender, ethnicity, and latency (case–control study design)

Subgroup	Preexisting asthma in patients with HS *N* (%) (*N* = 6748)*	Preexisting asthma in controls *N* (%) (*N* = 33,151)*	OR (95%CI)	Univariate *P* value
All	646 (9.6%)	2288 (6.9%)	**1.43 (1.30–1.57)**	** < 0.001**
Age, years^a^				
≥ 30	264 (7.8%)	903 (5.4%)	**1.48 (1.28–1.71)**	** < 0.001**
< 30	382 (11.3%)	1385 (8.4%)	**1.40 (1.24–1.57)**	** < 0.001**
Gender				
Male	246 (9.1%)	938 (7.0%)	**1.32 (1.14–1.53)**	** < 0.001**
Female	400 (9.9%)	1350 (6.8%)	**1.50 (1.34–1.69)**	** < 0.001**
Ethnicity				
Jews	569 (10.2%)	2029 (7.4%)	**1.42 (1.28–1.56)**	** < 0.001**
Arabs	77 (6.6%)	259 (4.4%)	**1.52 (1.17–1.98)**	**0.002**
Latency after the diagnosis of asthma				
0–5 years	64 (0.9%)	225 (0.7%)	**1.40 (1.06–1.85)**	**0.017**
6–10 years	162 (2.4%)	617 (1.9%)	**1.30 (1.09–1.55)**	**0.004**
> 10 years	386 (5.7%)	1340 (4.0%)	**1.44 (1.28–1.62)**	** < 0.001**

^*^Excluding patients with a diagnosis of asthma after HS or recruitment

^a^cutoff age was set at 30 since it represent the median age of study participants

*HS* hidradenitis suppurativa, *OR* odds ratio, *N* number, *C* confidence interval

Bold: significant value

We then conducted a multivariate analysis adjusting for putative confounders. The odds of HS after asthma persisted after adjusting for age and sex (age- and sex-adjusted OR, 1.43; 95% CI 1.30–1.57; P < 0.001) as well as for age, sex, ethnicity, BMI, and comorbidities (fully adjusted OR 1.41; 95% CI 1.27–1.55; *P* < 0.001).

### The risk of asthma in patients with hidradenitis suppurativa (retrospective cohort design)

Patients with HS and controls were cumulatively followed for 34,272.3 and 173,125.8 person-years, respectively. During this follow-up period, the incidence rate of asthma was estimated at 9.0 (95% CI 2.5–6.8) and 6.2 (95% CI 5.1–7.5) cases per 10,000 person-years among patients with HS and controls, respectively (Table 3).

**Table 3:** Incidence rates and hazard ratio of new-onset asthma among patients with hidradenitis suppurativa (cohort study design)

	HS (*N* = 6133)*	Control (*N* = 30,971)*
Follow-up time, PY	34,272.3	173,125.8
Median follow-up time, years (range)	6.1 (0.1–18.7)	6.1 (0.1–18.7)
Number of new-onset asthma events	31	108
Incidence rate / 10,000 PY (95% CI)	9.0 (6.3–12.7)	6.2 (5.1–7.5)
Unadjusted HR (95% CI) [*P* value]	1.45 (0.97–2.16) [0.069]	Reference
Sex-, age-, and ethnicity-Stratified analysis		
Male-specific HR (95% CI) [*P* value]	1.69 (0.85–3.34) [0.132]	Reference
Female-specific HR (95% CI) [*P* value]	1.34 (0.82–2.20) [0.240]	Reference
≥ 30 years-specific HR (95% CI) [*P* value]^a^	1.66 (0.96–2.86) [0.071]	Reference
< 30 years-specific HR (95% CI) [*P* value]^a^	1.26 (0.70–2.26) [0.439]	Reference
Jews-specific HR (95% CI) [*P* value]	1.38 (0.89–2.15) [0.152]	Reference
Arabs-specific HR (95% CI) [*P* value]	1.81 (0.72–4.60) [0.210]	Reference
Multivariate adjusted analysis		
Age- and sex-Adjusted HR (95% CI) [*P* value]	1.45 (0.97–2.16) [0.069]	Reference
Fully adjusted HR (95% CI) [P value]^b^	1.53 (0.98–2.38) [0.062]	Reference

*HS,* hidradenitis suppurativa; *HR,* hazard ratio; *CI,* confidence interval; *PY,* person-year

^*^Excluding patients with a diagnosis of asthma before HS or recruitment

^a^cutoff age was set at 30 since it represent the median age of study participants

^b^Adjusted for age, sex, ethnicity, BMI, and comorbidities (per modified CCI)

Bold: significant value

**Fig. 1:**
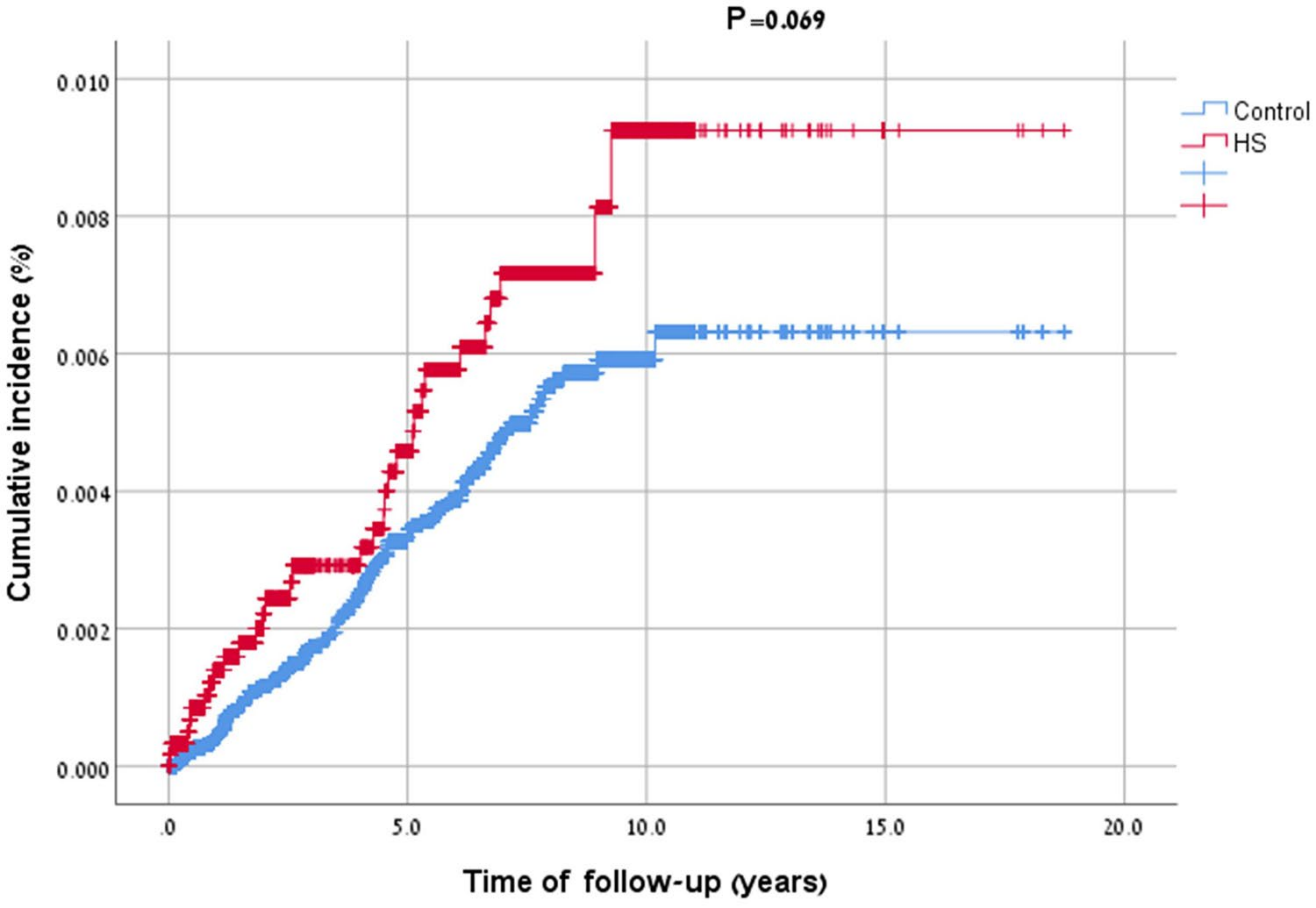
Kaplan–Meier curves demonstrating the cumulative incidence of asthma among patients with HS and controls

Taken together, the unadjusted risk of asthma was comparable in cases and controls (HR 1.45; 95% CI 0.97–2.16; *P* = 0.069; Fig. 1). In a stratified analysis, HS was not associated with an increased risk of asthma in all age, sex, and ethnicity categories (Table 3). The risk of asthma in HS fell shortly out of significance after controlling for age and sex (age- and sex-adjusted HR 1.45; 95% CI 0.97–2.16; *P* = 0.069) as well as for age, sex, ethnicity, BMI, and comorbidities (fully adjusted HR, 1.53; 95% CI 0.98–2.38; *P* = 0.062; Table 3).

### Features of patients with hidradenitis suppurativa and comorbid asthma

Compared to other patients with HS, those with HS and comorbid asthma were significantly younger at the onset of HS (30.7 [14.7] vs. 33.3 [15.1] years; *P* < 0.001) and had a higher frequency of Jewish ethnic background (OR 1.56; 95% CI 1.22–1.97; *P* < 0.001; Table 4). BMI, modified CCI, and the lifetime prevalence of diabetes mellitus, hypertension, hyperlipidemia, and smoking were distributed equally between the subgroups (Table 4).

**Table 4:** Characteristics of patients with hidradenitis suppurativa and comorbid asthma relative to the remaining patients with hidradenitis suppurativa

	HS with comorbid asthma (*n* = 677)	HS without asthma (*n* = 6102)	OR	95% CI	*P* value
Age at the onset of HS, years; mean (SD)	30.7 (14.7)	33.3 (15.1)	**0.88**^**a**^	**0.83–0.93**	** < 0.001**
Female sex, *n* (%)	420 (62.0%)	3651 (59.8%)	1.10	0.93–1.29	0.266
Jewish ethnicity, *n* (%)	594 (87.7%)	5013 (82.2%)	**1.56**	**1.22–1.97**	** < 0.001**
Body mass index; Kg/m2, mean (SD)^b^	27.9 (7.3)	27.6 (21.5)	1.00^b^	0.99–1.01	0.681
Modified Charlson Comorbidity index; mean (SD)^d^	0.4 (1.0)	0.4 (1.1)	0.96^c^	0.89–1.04	0.355
Diabetes mellitus, *n* (%)	62 (9.2%)	636 (10.4%)	0.87	0.66–1.14	0.304
Hypertension, *n* (%)	80 (11.8%)	773 (12.7%)	0.92	0.72–1.18	0.526
Hyperlipidemia, *n* (%)	177 (26.1%)	1795 (29.4%)	0.85	0.71–1.02	0.075
Smoking, *n* (%)	367 (54.2%)	3224 (52.8%)	1.06	0.90–1.24	0.497

Change in OR per 10 years ^a^, mg/kg^2^ unit ^b^ or point in CCI score^c^, ^d^excluding pulmonary diseases

*HS* hidradenitis suppurativa, *OR* odds ratio, *CI* confidence interval, *PY* person-year, *SD* standard deviation

Bold: significant value

We then assessed the overall survival rates of patients with HS and comorbid asthma relative to the remaining patients with HS. The risk of all-cause mortality was comparable between the two subgroups both in univariate (HR 0.94; 95% CI 0.49–1.80; *P* = 0.849; *Supplementary figure*) and the fully adjusted multivariate model (HR 0.86; 95% CI 0.44–1.68; *P* = 0.660).

## Discussion

This population-based study sheds light on the bidirectional association between HS and asthma. Our findings suggest that a patient with a history of asthma is more susceptible to developing HS than a non-asthmatic patient. However, based on our study, a diagnosis of HS does not confer an increased risk of developing asthma.

The comorbidity of HS with asthma was scarcely reported in the literature. In a case series depicting the characteristics of 11 patients with coexistent HS and pyoderma gangrenosum, two (18.2%) patients had comorbid asthma [16]. A chart review of 73 pediatric patients with HS, asthma, and other reactive airway diseases was detected among 19.2% of the study population [18]. In a recent trajectory analysis of a large group of patients with HS, asthma was found to precede the diagnosis of HS [19]. Our findings align with the observation of Kjærsgaard Andersen et al. [19] in view of the fact that in 95% of patients with both conditions, asthma preceded HS. While these studies contributed to evaluating the absolute burden of asthma among patients with HS, they lacked control groups and were underpowered to delineate the relative odds and risk of asthma in HS. A recent cross-sectional study revealed an inverse association between HS and allergic contact dermatitis, allergic rhinitis, and allergic conjunctivitis [15]. However, the association of HS with asthma was not evaluated in this study.

A deeper look into the pathogenesis of HS and asthma might contribute to interpreting the association between both conditions. Asthma is characterized by the presence of airway inflammation stemming from sensitization to inhaled allergens or irritants. Allergens are recognized by dendritic cells that induce Th2 cell proliferation and release of Th2 cytokines (IL-4, IL-5, and IL-13) [20]. A growing body of evidence has accumulated to suggest the involvement of Th17 cytokines (IL-17A, IL-17F, and IL-22) in the pathogenesis of neutrophilic asthma [21–23].

Utilizing proteomic and transcriptomic approaches to investigate the inflammatory response in HS, it has been shown that the immune response in HS relies heavily on IFN-γ, IL-36, and TNF, with a considerable contribution of IL-17A to the initiation of skin inflammation [24, 25]. Lima et al. [26] found that IL-17 is upregulated in perilesional skin of HS patients and that neutrophils participate in the positive feedback loop releasing IL-17, thereby sustaining the inflammatory process in HS. The shared pathogenic role of Th-17-mediated immunity might pave the way to interpret the observed association of HS with a history of asthma.

Utilizing two different epidemiological approaches, the current study was powered to investigate the bidirectional relationship between HS and asthma. To the best of our knowledge, this association has not been sufficiently investigated in a controlled population-based manner. The large sample size and the comprehensiveness of the database argue against the existence of meaningful selection bias and substantiate the generalizability of our observations. However, the current study has some limitations to acknowledge. Relying on routinely documented clinical data (retrieved from a database for clinical purposes) interfered with delineating the severity scores of HS and the morphological features of eligible patients. The relatively small number of positive outcomes in the retrospective cohort design might have statistically attenuated the analysis. Given the ethnically homogeneous nature of the study population, further studies originating from other ethnic groups are warranted to reproduce our findings.

The current study depicts that a history of asthma confers increased susceptibility to HS. Patients with HS, on the other hand, experience a comparable risk of developing subsequent asthma. The presence of comorbid asthma among patients with HS is associated with an earlier onset of HS but does not influence the all-cause mortality of patients. Physicians managing patients asthma should be aware of these findings and increased risk for HS. The diagnostic threshold of HS should be lowered in patients with a history of asthma presenting with painful intertriginous nodules. The pathomechanism underlying this epidemiological finding should be further investigated. It remains to be investigated whether an early asthma treatment has the potential to mitigate the risk of HS development.

### Supplementary Information

Below is the link to the electronic supplementary material.

Supplementary file1 (JPG 175 KB)
