# Supplementary figures and images for: A history of asthma is associated with susceptibility to hidradenitis suppurativa: a population-based longitudinal study
Source: Arch Dermatol Res. 2023 Aug 29;315(10):2845–51. doi: 10.1007/s00403-023-02693-4 (PMC10615903; doi:10.1007/s00403-023-02693-4)

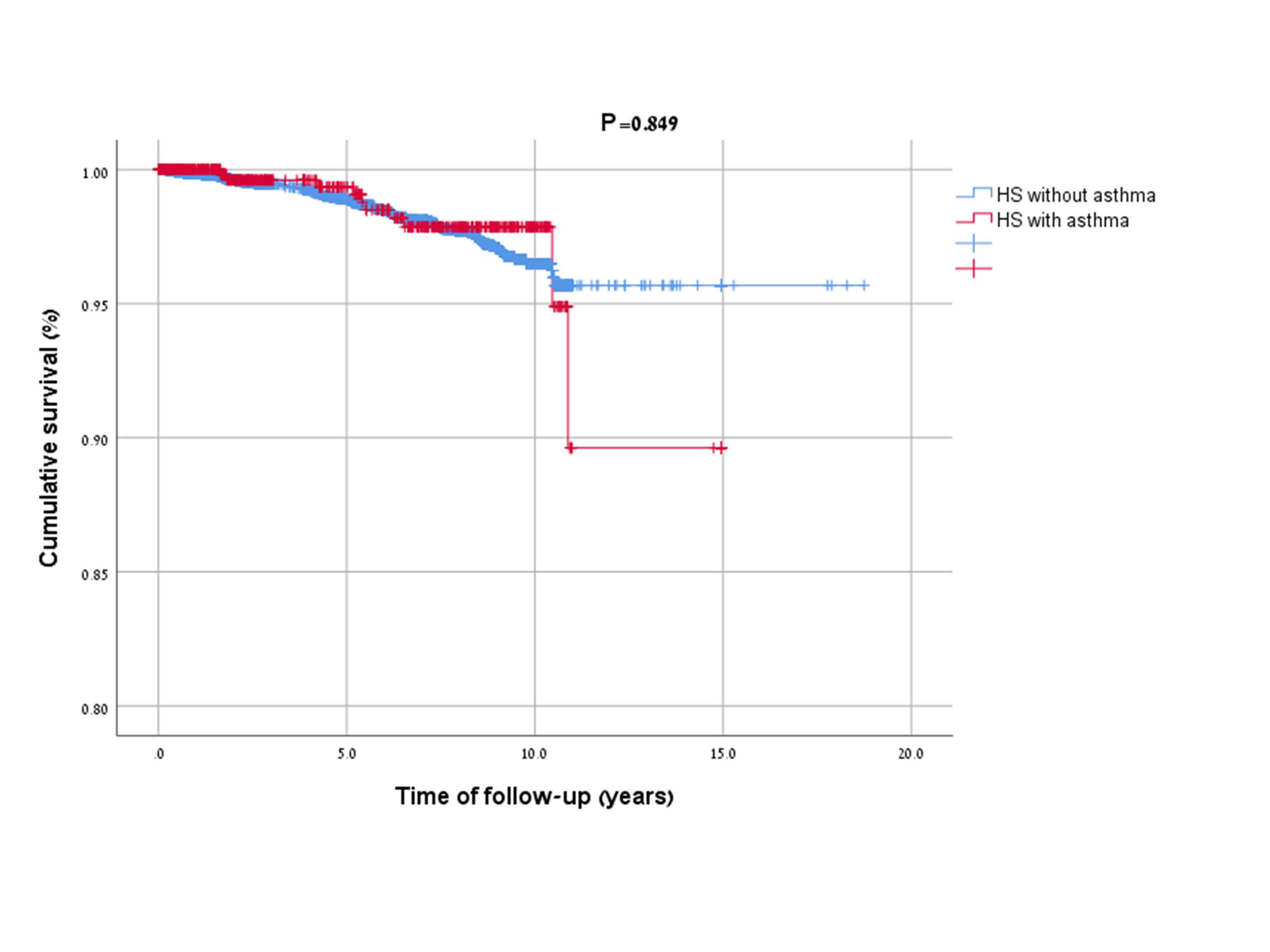

Supplement: Supplementary file 1 — Supplementary file1 (JPG 175 KB) [file 403_2023_2693_MOESM1_ESM.jpg]
